# Production of haploids and doubled haploids in oil palm

**DOI:** 10.1186/1471-2229-10-218

**Published:** 2010-10-07

**Authors:** Jim M Dunwell, Mike J Wilkinson, Stephen Nelson, Sri Wening, Andrew C Sitorus, Devi Mienanti, Yuzer Alfiko, Adam E Croxford, Caroline S Ford, Brian P Forster, Peter DS Caligari

**Affiliations:** 1 School of Biological Sciences, University of Reading, Whiteknights, Reading, RG6 6AS, UK; 2 Institute of Biological, Environmental and Rural Sciences, Aberystwyth University, Aberystwyth, SY23 3DA, UK; 3 Sumatra Bioscience Pte Ltd, 8 Eu Tong Sen Street, #16-94/95 The Central, 059818, Singapore; 4 PT Sumatra Bioscience, Bah Lias Research Station, North Sumatra, Indonesia; 5 BioHybrids International Ltd, Earley, Reading, RG6 5FY, UK; 6 Instituto de Biología Vegetal y Biotecnología, Universidad de Talca, 2 Norte 685, Talca, Chile

## Abstract

**Background:**

Oil palm is the world's most productive oil-food crop despite yielding well below its theoretical maximum. This maximum could be approached with the introduction of elite F_1 _varieties. The development of such elite lines has thus far been prevented by difficulties in generating homozygous parental types for F_1 _generation.

**Results:**

Here we present the first high-throughput screen to identify spontaneously-formed haploid (H) and doubled haploid (DH) palms. We secured over 1,000 Hs and one DH from genetically diverse material and derived further DH/mixoploid palms from Hs using colchicine. We demonstrated viability of pollen from H plants and expect to generate 100% homogeneous F_1 _seed from intercrosses between DH/mixoploids once they develop female inflorescences.

**Conclusions:**

This study has generated genetically diverse H/DH palms from which parental clones can be selected in sufficient numbers to enable the commercial-scale breeding of F_1 _varieties. The anticipated step increase in productivity may help to relieve pressure to extend palm cultivation, and limit further expansion into biodiverse rainforest.

## Background

Success of early F_1 _hybrid maize varieties exemplifies the advantages of heterosis [1]. The use of doubled haploids as parents for F_1 _variety production fully exploits this phenomenon and has enabled substantial yield improvements in several crops [2,3]. This strategy was outlined with the first DH crop variety [4] and has led to H/DH production systems being described for > 250 species [5]. However, few of these protocols generate the large numbers of Hs/DHs needed for commercial breeding, with just three methods (androgenesis, wide crossing, gynogenesis [6]) routinely adopted for H/DH production in only 30 species [5]. The most important of these methods in widespread use in commercial breeding is the generation of haploids in maize via pollination with a haploid inducing line such as a 'Stock 6' derivative. Desire for a more generic H/DH production system to improve agricultural yields is increasing as population growth, climate change, biofuel demand and other land-use pressures intensify. Clearly, in any species the production of F_1 _varieties depends not only on the production of homozygous lines to act as parents, but also it requires an efficient method to intercross the parents. This latter procedure is relatively simple in species with an outcrossing breeding system, like maize or oil palm, compared with those with an inbreeding system like rice or wheat. Production of F_1 _hybrids has been achieved successfully in this category of crops (for example hybrid rice in China) but often requires a male sterility system.

Annually, oil palm (*Elaeis guineensis*) yields eight to ten times more oil per hectare than rapeseed or soybean [7,8] and in 2008 generated 38.9 million tonnes of oil worldwide [9]. The area assigned to the crop expanded ~1.7 fold between 1997 (8.7 M ha) and 2007 (14.6 M ha) [9] with further increases forecast. Over this same period global production of palm oil increased ~2.2 fold from 18 to 38.9 Mt y^-1^. Thus, yield increases have been achieved predominantly by expansion of cultivated area and not through yield enhancement. This trend raises concerns over the ecological impact of felling rainforest to accommodate oil palm cultivation [10,11] and has stimulated debate over strategies to limit further agricultural expansion [12-14]. One option explored here is to use market forces to help address the problem. If F_1 _varieties could increase yields sufficiently to exceed demand, commodity prices would fall. This would discourage clear felling and simultaneously incentivise early replacement of existing plantations with high-yielding varieties. Feasibility of the approach clearly relies on the ability to gain marked improvements in yield. Current yields of oil palm (generally 4-10.5 t ha^-1^) [15,16] are much lower than the most conservative estimates of the crop's potential (17 t ha^-1 ^[14] to 60 t ha^-1 ^[16]). Indeed, yields per hectare in the two largest producer countries (Indonesia and Malaysia) have remained static for 30 years [9]. It should be noted, however, that in both these countries there are examples of selected varieties with much higher yields, with the highest yields from commercial breeding trials already exceeding 10 t ha^-1^.

To date, a H/DH-derived F_1 _breeding approach has been precluded by the repeated failure to secure H/DHs via anther or microspore culture [17] and successful generation of H/DHs in oil palm is unreported in the literature. The report of a spontaneous H in the related coconut palm [18] and in other species [19] nevertheless gave hope that spontaneous Hs may also occur in oil palm. However, the characteristically rare occurrence of spontaneous H/DHs necessitates development of an effective high-throughput screening system. Phenotypic characteristics of H/DH (slow growth, altered flowering phenology, smaller stomata and smaller organs [5]) could be used for diagnosis but are difficult to score qualitatively on a large scale and require plants of a reasonable size. An alternative strategy is to seek undefined atypical phenotypic features that may arise from reduced cell size and/or the hemizygous state of haploid individuals (homozygous for DHs) and that are manifest at the seedling stage when high-throughput visual assessment is more plausible. A more directed approach is also possible. Spontaneous H/DH seedlings are often associated with aberrant germination features, such as twin embryos from the same carpel [20], providing a defined feature for phenotypic selection. Here, we combined a large-scale visual survey for undefined atypical palm seedling phenotypes coupled with active selection for seeds with twin embryos to assemble a sub-population of seedlings enriched for H/DHs.

## Results

**Figure 1:**
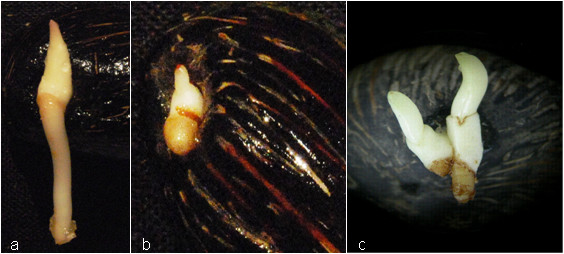
**Seed germination morphology for H/DH identification**. a: normal; b: abnormal; c: twin embryo.

**Figure 2:**
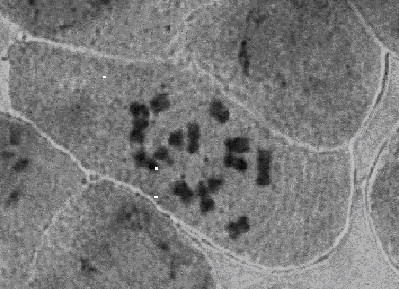
**Chromosome spread of a haploid root cell from oil palm containing 16 C-metaphase chromosomes**.

Over two years, we performed two large-scale screens for morphological 'off-types' among oil palm seedlings generated by the Bah Lias Research Station, Indonesia. The first screen utilised 10,900,000 seedlings from a wide range of crosses and identified 3,854 morphological 'off-types' (H/DH candidates), of which 53 had twin embryos and 3,801 were phenotypically abnormal (Figure 1). The second screen of approximately 10,000,000 seedlings from commercial seed production activities and approximately 1,000,000 seedlings from breeding experiments generated 5,704 H/DH candidates, of which 5,601 were phenotypically abnormal and 103 had twin embryos. More than 2,000 of these seedlings (including all those with twin embryos) were transferred to the nursery prior to further screening. Although Hs could be identified relatively easily on the basis of their reduced genome size, we initially wished to target the more difficult, but more valuable DHs to circumvent the need for chromosome doubling. For the second level screen, we exploited the fact that Hs and DHs would be either hemi- or homozygous across all loci; thus individuals exhibiting heterozygosity at any locus could be discarded. Applying this logic, we performed a sequential screen using 9-15 microsatellite markers (Table 1) on all individuals and found 117 seedlings that exhibited a single allele across all loci (Table 2). These individuals were retained as candidate H/DH, and subsequent flow cytometry of leaf samples identified 83 as H, and 34 as diploid (Table 2). The haploid status of six palms was further confirmed by cytological examination of intact cells from root squashes. Each contained the expected 16 chromosomes (Figure 2).

**Table 1:** Microsatellite primer pairs used to identify homozygous DH or hemizygous H candidates in the initial molecular screen.

**No**.	Forward primer (5'-3')	Reverse primer (5'-3')
1	GAGATTACAAAGTCCAAACC	TCAAAATTAAGAAAGTATGC
2	ACGCATGCAGCTAGCTTTTC	CGCGTGAAAGATATGAATCAAC
3	CACGCACGCAGTTTATTCTT	GGATGTATGCTTTACCTCCGAAT
4	CCCCTTTTGCTTCCCTATTT	CTCCTTTTCCCCATCACAGA
5	GACACAAGCAAAAACAAAAGCA	ATTCTGAAAGGAGGGGGAAA
6	ATATGTGTGGGTGTGCGTGT	TGCCTCTGGTTGTTAGTCTGG
7	TCTCTCTCTCTCTCTCTATGTGTGTGT	TGGCAATCAGCACACATTCT
8	GCAGCTCTTTCCACACCTCT	TGTGGTCTCCTGAGGAAGATG
9	TTTTCCCCATCACAGAATTG	CCCCTTTTGCTTCCCTATTT
10	TAGCCGCACTCCCACGAAGC	CCAGAATCATCAGACTCGGACAG
11	AGCTCTCATGCAAGTAAC	TTCAACATACCGTCTGTA
12	CCTTCAAGCAAAGATACC	GGCACCAAACACAGTAA
13	GTAGCTTGAACCTGAAA	AGAACCACCGGAGTTAC
14	GCTCGTTTTTGTTTAGGTGA	TTTTCTCCATAGTCCGTTAC
15	CCTCGGGTTATCCTTTTTACC	TGGCTGGCTTCGGTCTTAG

Markers 10-15 obtained from Billotte *et al. *[27].

**Table 2:** Results of ploidy analysis by flow cytometry of 117 candidate H/DH palms identified as both morphologically atypical and homozygous for the markers listed in Table 1.

Candidate	DNA sample code	No. markers used	Ploidy
50-Mix5-7	11260406301	9	x
50-03060367C	07280501801	15	x
50-03060260C-2	07280501901	15	x
53-03080954C-2	09270500101	10	x
53-03090761C-5	09280504501	10	x
BATCH 51;03060318C;1	060728_0010_01_a	15	x
BATCH 53;03090761C;5	060728_0018_01_a	15	x
0623/172;05095508C;1	060728_0021_01_a	15	x
BATCH 50;03060260C;2	060728_0027_01_a	15	x
0611/32;05050248C;1	060728_0032_01_a	15	x
0611/16;05050228C;1	060728_0034_01_a	15	x
BATCH 53;03080954C;2	060728_0035_01_a	15	x
06 412;04059061B;3	060728_0050_01_a	14	2x
0628/152;05100720C;1	060729_0021_01_a	15	x
0628/185;05100351C;1	060729_0063_01_a	15	x
BATCH 51;03060626C;1	060729_0127_02_a	15	x
BATCH 67;0409034MC;2	060729_0130_02_a	14	2x
BATCH 67;0409034MC;4	060729_0131_02_a	15	2x
BATCH 67;0409034MC;15	060729_0132_02_a	15	2x
BATCH 65;0409034MC;7	060729_0134_02_a	15	2x
BATCH 65;0409034MC;35	060729_0138_02_a	15	2x
BATCH 65;0409034MC;56	060729_0139_02_a	15	2x
BATCH 65;0409034MC;50	060729_0141_02_a	15	2x
BATCH 65;0409034MC;47	060729_0142_02_a	15	2x
0628/53;05090595C;1	060731_0043_01_a	15	x
0627/125;05090717C;2	060731_0065_01_a	15	x
0627/12;05080220C;1	060731_0080_01_a	15	x
0627/6;05080095C;1	060731_0086_01_a	14	x
0631/Normal;05039033B;31	060731_0265_01_a	14	x
64-0409021MC-34	02130604301	15	2x
64-0410040MC-1	02130604801	15	2x
51-03060626C	02130605301	15	x
64-0410040MC-20	02140600401	15	2x
64-0410040MC-16	02140600801	15	2x
65-0409021MC-2	02140601001	15	2x
06 412B-04059061B-3	02170605501	15	2x
06 412B-04129091B	02170605801	15	2x
0550-15/05010827C	02200602401	15	x
0550-17/05010442C-1	02200602601	15	x
0550-23/05020059C	02200603101	15	x
0550-33/05020568C	02200603401	15	x
0550-36/05020420C-2	02200603701	15	x
0550-40/05010880C	02200607501	14	x
0551-36/05020511C	02200607601	15	x
0551-32/05020361C-1	02210600401	15	x
0552-4/05010836C-2	02210600901	15	x
0552-38/05020501C	02210603101	14	x
0552-39/05020415C	02210603201	15	x
0552-31/05020858C	02210603701	15	x
0552-91/05020375C	02210603901	15	x
0552-111/05020626C	02210607201	15	x
0552-128/05020558C-1	02210607701	15	x
0601-35/05020946C	02210608201	15	x
0601-42/05030201C-6	02210609501	15	x
0601-51/05030224C-2	02220600201	15	x
0607-21/05040317C-3	02220601801	14	x
0606-32/05040240C	02220606201	13	x
0601-77/05020961C	02230600701	15	x
0601-62/05030147C	02230601401	15	x
0601-54/05030462C	02230601901	15	x
0551-21/05020271C-1	02200605801	14	x
0601-9/05020843C-2	02230603101	15	x
0602-17/05020631C-1	02230605501	15	x
0607-111/05040970C-1	03010600201	15	x
0607-81/05040578C-1	03010600501	15	x
0607-73/05040573C-1	03010605101	15	x
0607-89/05040748C-3	03010605501	15	x
0607-102/05050016C-2	03010606601	15	x
0608-15/05040519C-3	03010606901	15	x
0608-45/05041003C-1	03150603401	15	x
0610-60/05041024C-2	03150604401	15	x
0610-124/05055039C-1	03150604601	15	x
0609-54/05050089C-2	03150604701	15	x
0610-41/05050352C-1	03150606701	15	x
0609-58/05050255C-1	03220600201	15	x
0610-82/05050099C-2	03220601401	15	x
0610-77/05050353C-1	03220602701	15	x
0610-121/05055090C-1	03220603301	15	x
0610-81/05050099C-1	03220605901	15	x
0609-100/05055311C-1	03290600301	15	x
0610-11/05040938C-1	03290601101	15	x
0610-68/05050376C-3	03290602001	15	x
0610-58/05050344C-1	03290602201	15	x
0610-73/05050594C-3	03290603301	15	x
0611-84/05050714C-4	03290605001	15	x
0611-70/05050223C-1	03290606701	15	x
0611-73/05050351C-1	03290608001	15	x
0610-67/05050376C-2	04050600501	15	x
0610-40/05050102C-2	04050600901	15	x
0611-99/05050544C-1	04050602601	15	x
0611-110/05055011C-1	04050603601	15	x
0612-2/05050017C-1	04050609101	15	x
0612-70/05050530C-1	04050609201	15	x
0612-76/05050512C-1	04050610301	15	x
0611-109/05055144C-1	04120600101	15	x
0611-31/05050220C-1	04120600601	15	x
0611-38/05050284C-4	04120600901	15	x
0611-40/05050171C-1	04120601101	14	x
0612-80/05050713C-1	04120603101	15	x
65-0409034 MC-66	060829_0001_02_a	15	2x
65-0409034 MC-68	060829_0002_02_a	15	2x
65-0409034 MC-72	060829_0003_02_a	14	2x
65-0409034 MC-111	060829_0005_02_a	15	2x
65-0409034 MC-94	060829_0011_02_a	14	2x
65-0409034 MC-120	060829_0012_02_a	15	2x
65-0409034 MC-144	060829_0013_02_a	15	2x
65-0409034 MC-133	060829_0015_02_a	15	2x
65-0409034 MC-187	060829_0020_02_a	15	2x
65-0409034 MC-193	060829_0021_02_a	14	2x
65-0409034 MC-199	060829_0023_02_a	15	2x
65-0409034 MC-135	060829_0025_02_a	15	2x
65-0409034 MC-114	060829_0026_02_a	13	2x
65-0409034 MC-147	060829_0027_02_a	15	2x
65-0409034 MC-36 B	060829_0030_02_a	15	2x
65-0409034 MC-39 A	060829_0031_02_a	15	2x
65-0409034 MC-73 A	060829_0034_02_a	15	2x
65-0409034 MC-71 A	060829_0035_02_a	14	2x

Note: in this initial round, no DH was found. The DH (0644-219/05049582C) was detected in a subsequent batch.

A larger-scale survey for heterozygosity was then performed using 97 additional microsatellites (Table 3) to confirm absolute hemizygosity of Hs and identify 'false' candidate DHs showing any heterozygosity. All Hs produced single-allele peak profiles across all microsatellites, thereby discounting fixed heterozygosity via locus duplication for all markers used. All diploids were heterozygous at several loci and so discarded. However, one diploid (0644-219/05049582C) identified from a later screen (see below) was homozygous across all 36 mapped loci found to be heterozygous in the maternal parent (palm number BL013/12-06). Taking account of linkage between mapped markers, the probability of such an individual occurring by chance following selfing was 8.72 × 10^-8 ^(see Methods). This palm was therefore deemed a spontaneous DH.

**Table 3:** Microsatellite markers (described by Billotte *et al. *[27]) used for a larger-scale survey for hemizygosity of Hs and homozygosity of DH candidates previously identified by the morphological screen, microsatellite pre-screen (15 markers) and flow cytometry screen.

**No**.	Forward primer (5'-3')	Reverse primer (5'-3')
16	GACCTTTGTCAGCATACTTGGTGTG	GCAGGCCTGAAATCCCAAAT
17	ATGCATGTGATTTTATTAGGTGAGA	CGACCCTCAGTCAATCAGTAAG
18	AAGCTAGCGACCTATGATTTTAGA	AAACAAGTAATGTGCATAACCTTTC
19	CCCACCACCCCTAGCTTCTC	ACCCCGGTCCAAATAAAATC
20	AGAGAGAGAGAGTGCGTATG	GTCCCTGTGGCTGCTGTTTC
21	GGGTAGCAAACCTTGTATTA	ACTTCCATTGTCTCATTATTCT
22	CGAGGCCCAAAAACATTCAC	GGTCCCGATCCCGTCTACTG
23	TTGCGGCCCATCGTAATC	TCCCTGCAGTGTCCCTCTTT
24	AGGGAATTGGAAGAAAAGAAAG	TCCTGAGCTGGGGTGGTC
25	AGCAAGAGCAAGAGCAGAACT	CTTGGGGGCTTCGCTATC
26	TAGCCATGCCGCCACCACTT	CAATCCATTAGCGTGCCCTTCT
27	CTTACCCCGCCTCCTCTCCT	CGAAATGCCCTTCCTTTACACTA
28	CCTTATATCGCACGGGTTCC	TTCTTGGGGTCTCGCTACGG
29	GCAAGATGCAATGGAGTTCA	CAAACCGCAGCAAGTCAGA
30	GCAAAATTCAAAGAAAACTTA	CTGACAGTGCAGAAAATGTTATAGT
31	CGTTCATCCCACCACCTTTC	GCTGCGAGGCCACTGATAC
32	GAATGTGGCTGTAAATGCTGAGTG	AAGCCGCATGGACAACTCTAGTAA
33	ACATTCCCTCTATTATTCTCAC	GTTTTGTTTGGTATGCTTGT
34	AAGCCAACTTCACAGATATGTTGAT	ATGAGCCTAACAAAGCACATTCTAA
35	AGTGAGGTATGGTTGATTAGGA	TATTGATAGCATTTGGGATTAG
36	CTCCGATGGTCAAGTCAGA	AAATGGGGAAGGCAATAGTG
37	GCCGTTCAAGTCAATTAGAC	TTTGGGAGCAAGCATTATCA
38	TGCTTCTTGTCCTTGATACA	CCACGTCTACGAAATGATAA
39	CACCACATGAAGCAAGCAGT	CCTACCACAACCCCAGTCTC
40	TTTTATTTTCCCTCTCTTTTGA	ATTGCGTCTCTTTCCATTGA
41	CATATGGCGCACAGGCAC	GCAATACAAGAGCACCCAAAT
42	AGTTGGTTTGCTGATTTG	TGTTGCTTCTTTGATTTTC
43	GCTGAAGATGAAATTGATGTA	TTCAGGTCCACTTTCATTTA
44	ATGACCTAAAAATAAAATCTCAT	ACAGATCATGCTTGCTCACA
45	GGTGCAAGAGAGGAGGAATG	TTTGGTAGTCGGGCGTTTTA
46	GTTTGGCTTTGGACATG	TCCATCACAGGAGGTATAG
47	TGTTTTGTTTCGTGCATGTG	GGCTGACATGCAACACTAAC
48	CGGTTTTGTCGCATCTATG	GTCGTCAGGGAACAACAGT
49	CAATCATTGGCGAGAGA	CGTCACCTTTCAGGATATG
50	GAGCATGACGCAAACAAAGG	GCAACATGTTTGATGCATTAATAGTC
51	TCCAAGTAGCAAATGATGAC	TGCCCTGAAACCCTTGA
52	GAAGGGGCATTGGATTT	TACCTATTACAGCGAGAGTG
53	AACACTCCAGAAGCCAGGTC	GGTTTAGGTATTGGAACTGATAGAC
54	GATCCCAATGGTAAAGACT	AAGCCTCAAAAGAAGACC
55	TGTGGTTTGAGGCATCTTCT	GCCCACCAAAAGAAAGTAGT
56	TAGCCGCACTCCCACGAAGC	CCAGAATCATCAGACTCGGACAG
57	TCAAAGAGCCGCACAACAAG	ACTTTGCTGCTTGGTGACTTA
58	GGGGATGAGTTTGTTTGTTC	CCTGCTTGGCGAGATGA
59	TCTAATGCTCCCAAGGTACA	GGCTTGGTCCACGATCTT
60	AGCTCTCATGCAAGTAAC	TTCAACATACCGTCTGTA
61	TCCTCACTGCTCCTCTAATC	ACTCCCTATGGACCTTAGTC
62	AGGGAGGCGAACGAGAAACA	CGACTGCTGATGGGGAAGAG
63	CTACGGACTCACACCTATAT	ATGGTTCATCAATGAGATC
64	GTGAGCGATTGAGGGGTGTG	GGGGCTTGATTGAGTATTTCCA
65	AGGGCAAGTCATGTTTC	TATAAGGGCGAGGTATT
66	GAAGCCTGAGACCGCATAGA	TTCGGTGATGAAGATTGAAG
67	TTTCTTATGGCAATCACACG	GGAGGGCAGGAACAAAAAGT
68	GTTTATCATTTTGGGGTCAG	CGGTGTCCCTCAGGATGTA
69	CATGCACGTAAAGAAAGTGT	CCAAATGCACCCTAAGA
70	AATCCAAGTGGCCTACAG	CATGGCTTTGCTCAGTCA
71	TGTAGGTGGTGGTTAGG	TGTCAGACCCACCATTA
72	AGCAAGACACCATGTAGTC	GACACGTGGGATCTAGAC
73	AAAAGCCGATAGTGGGAACA	ATGCTGAGAGGTGGAAAATAGAG
74	GTCCATGTGCATAAGAGAG	CTCTTGGCATTTCAGATAC
75	AGCCAATGAAGGATAAAGG	CAAGCTAAAACCCCTAATC
76	CAATTCCAGCGTCACTATAG	AGTGGCAGTGGAAAAACAGT
77	GGGCTTTCATTTTCCACTAT	GCTCAACCTCATCCACAC
78	GACAGCTCGTGATGTAGA	GTTCTTGGCCGCTATAT
79	ACTTGTAAACCCTCTTCTCA	GTTTCATTACTTGGCTTCTG
80	CCTTCAAGCAAAGATACC	GGCACCAAACACAGTAA
81	CCACTGCTTCAAATTTACTAG	GCGTCCAAAACATAAATCAC
82	GGGAGAGGAAAAAATAGAG	CCTCCCTGAGACTGAGAAG
83	AGCAGGGCAAGAGCAATACT	TTCAGCAGCAGGAAACATC
84	GCCTATCCCCTGAACTATCT	TGCACATACCAGCAACAGAG
85	CATCAGAGCCTTCAAACTAC	AGCCTGAATTGCCTCTC
86	ATTCATTGCCATTCCCTTCA	TTGTCCCCTCTGTTCACTCA
87	ATTGCAGAGATGATGAGAAG	GAGATGCTGACAATGGTAGA
88	TCTCCCAAATCACTAGAC	ATCTGCAAGGCATATTC
89	ACGTTTTGGCAACTCTC	ACTCCCCTCTTTGACAT
90	TCCACTCTGGCAACTCC	AAGGATGGGCTTTGTAGT
91	TTTAGAGGACAAGGAGATAAG	CGACCGTGTCAAGAGTG
92	AGCAAAATGGCAAAGGAGAG	GGTGTGTGCTATGGAAGATCATAGT
93	GTAGCTTGAACCTGAAA	AGAACCACCGGAGTTAC
94	AAGCCACCAGGATCATC	GTCATTGCCACCTCTAACT
95	TTACTTGCTAAGCTCTCTAGC	TGGCTGTTTAATCTGTCTG
96	TCTATATTTGGTTGGCTTGA	ACTCATTTCAATCTCAGTGTC
97	TGCTACGTGCTGAAATA	ATTTCAGGTTCGCTTCA
98	CCTCCACTTCTCTTCATCTT	CTTCCTCAAGCTCAAACAAT
99	GATGTTGCCGCTGTTTG	CATCCCATTTCCCTCTT
100	ATGCTCCACCAAGTTTA	CACATCCTAGCATCATTG
101	AAGCAATATAGGTTCAGTTC	TCATTTTCTAATTCCAAACAAG
102	GCTCGTTTTTGTTTAGGTGA	TTTTCTCCATAGTCCGTTAC
103	CAGCACACAAATGACAT	CACCTTTCCTTTTTGTC
104	CCTATTCCTTACCTTTCTGT	GACTTACTATCTTGGCTCAC
105	CCTTGCATTCCACTATT	AGTTCTCAAGCCTCACA
106	CCTCCTTTGGAATTATG	GTGTTTGATGGGACATACA
107	ATTGGAGAGCACTTGGATAG	TTCTCTTCCTTCTCACTTGT
108	AGCCAGATGGAAATACAC	GTGCGATAAAGAGGAGAGT
109	TAGTTTTCCCATCACAGAGT	ACAATATTTAGACCTTCCATGAG
110	GTGCAGATGCAGATTATATG	CCTTTAGAATTGCCGTATC
111	ACAATAACCTGAGACAACAAGAAAC	ATACATCCCCTCCCCTCTCT
112	GAACTTGGCGTGTAACT	TGGTAGGTCTATTTGAGAGT

These initial screens collectively revealed 83 spontaneous Hs but no DHs (although one DH was discovered subsequently), with the undirected phenotypic 'off-type' selection proving substantially more effective than screening for twin embryos. This result suggests that our method could be used to secure large numbers of Hs but is less able to isolate DHs at useful frequencies. This finding, when coupled with the routine nature of H chromosome doubling in other crops [21], suggested the most promising route for commercial DH production lay in the isolation of Hs followed by somatic doubling. In subsequent screening of abnormal seedlings, high-throughput flow cytometry therefore replaced molecular analysis for candidate H identification. Haploid identity was then supported using at least 15 microsatellite markers. Plants identified as diploid by flow cytometry continued to be screened for DHs as above. Using this amended screening procedure, we have identified over 1,100 H palms from approximately 60 million seedlings (to July 2009).

**Figure 3:**
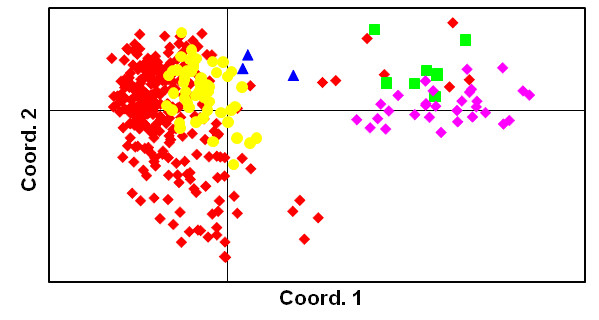
**Principal Coordinates Analysis Plot of 95 diploid and 27 haploid palms based on 28 microsatellites**. Red diamonds: haploids; green squares: commercial pisiferas; blue triangles: commercial teneras; yellow diamonds: commercial duras; purple diamonds: Ghanaian wild material. Microsatellite data in Table 7.

To have maximum utility this H/DH material should encompass as much genetic diversity from within the breeding germplasm as possible. A Principal Coordinates Analysis performed on H profiles using 28 microsatellite loci showed the first two axes accounted for 58% of the detected variation. While most Hs had a strong affinity to commercial duras, Hs have also been generated from pisifera types and overall variability amongst Hs encompassed that seen for the entire commercial palm material (Figure 3).

**Figure 4:**
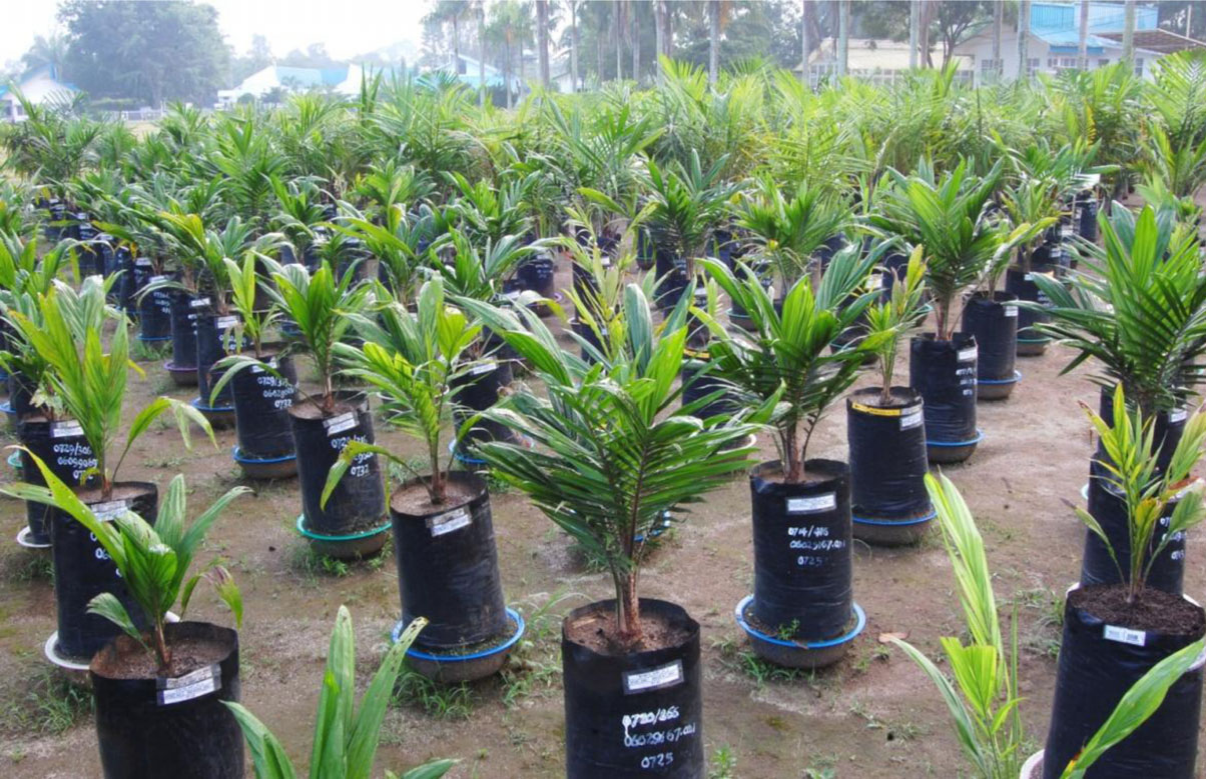
**Selection of haploid oil palm plants growing in a nursery**.

**Figure 5:**
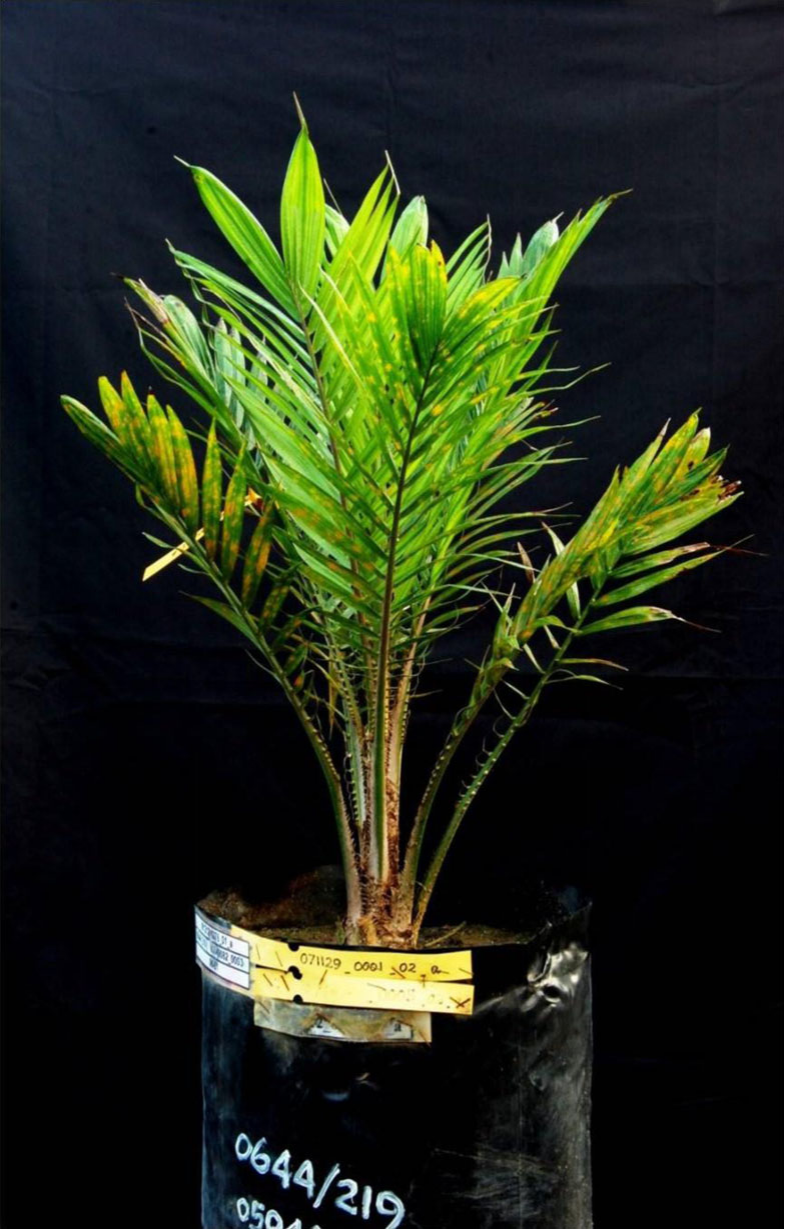
**Doubled haploid palm**.

Effort then focussed on the creation of DHs from this rich germplasm of H genotypes (Figure 4). The most direct route to obtain DHs is to use chemical application to induce chromosome doubling. We applied a range of treatments to 50 H seedlings and screened leaves of the recovered material for evidence of chromosome doubling. Flow cytometry revealed that 48 seedlings contained substantial diploid sectors in their leaves; one palm was 100% doubled after exposure to10 mM colchicine (Figure 5) and 100 ppm GA_3. _To date, 16 H genotypes have produced pollen. This finding demonstrates scope for securing fertile gametes from diploid inflorescences or inflorescence sectors for DH or F_1 _production. Indeed, seed set using pollen from DH material has now been achieved (data not shown). Whilst further optimization work is required, our results when combined with experience in other crops [21] suggest routine production of fertile DH oil palm lines will be a relatively simple task.

## Discussion and Conclusions

The simple high-throughput phenotypic-genotypic seedling selection system used here provides a fourth practical approach to supplement androgenesis, wide crossing and gynogenesis [6] and has potential for many crops where H/DH production remains elusive. The prospect of adopting a similar untargeted approach more widely seems both plausible and attractive, and may be possible without experienced operators, especially as sophisticated phenomic screening systems [22] become more accessible.

In the case of oil palm, the efficacy of our H screening combined with the demonstrated ability to create DH palms, opens the way for the development of 100% true-breeding parental clones for F_1 _variety breeding. Thereafter, it is hoped that the potential genetic gain available from oil palm F_1 _hybrids will match that in other crops. If such a gain is achieved it could be beneficial in several ways. First, high-yielding F_1 _palms are likely to accelerate replacement of palms in existing plantations and cause a step-increase in production. Secondly, this breeding strategy provides greater flexibility for breeders to respond rapidly to emergent threats (e.g. climate change). Thirdly, using palm oil and its associated wastes for energy generation [7] could substantially reduce carbon-based emissions currently associated with the palm oil lifecycle [23]. Fourthly, DH oil palms could be exploited in combination with transgenic techniques that are now available for this crop [24]. Looking forward, the clear challenge is to maintain and improve oil palm productivity in the face of a changing climate sufficient to keep pace with growing demand [25]. However, it is important to point out that breeding is simply one stage in a long process from plantation to the eventual processed product and the economic realities of this international industry will finally determine the impact of any novel technology on the global agricultural system for this crop.

The provision here of a system for haploid-based F_1 _hybrid breeding in oil palm represents the first technological breakthrough likely to lead to step improvements in yield for this crop, and can also be applied to other crops recalcitrant to *in vitro *based H/DH systems. This methodology, in particular the application of high-throughput flow cytometry, has recently been applied successfully to two other tropical crops, namely rubber (*Hevea brasiliensis *L.) and cocoa (*Theobroma cacao *L.) (Nasution et al. unpublished).

## Methods

**Figure 6:**
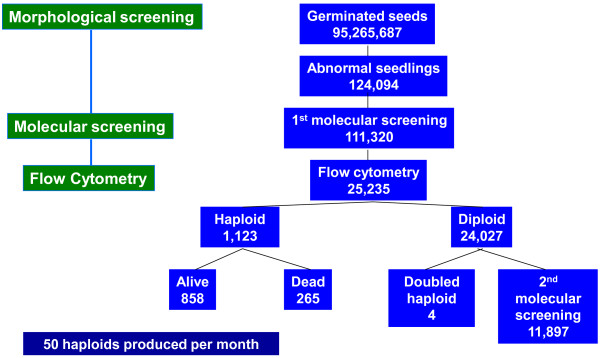
**Summary of stages for identification of haploid and doubled haploid palm**.

Hs and DHs were identified using three methods: a morphological screen; homozygosity/hemizygosity assessment; and ploidy level measurement. Initial screens emphasized identification of candidate DHs where seedling morphology screening was followed by homozygosity/hemizygosity assessment using microsatellites. H/DHs were then distinguished by flow cytometry and DHs subjected to an extensive homozygosity screen (Figure 6). As spontaneous DH frequency was low, later screens emphasized H recovery where the morphological screen was followed by flow cytometry; homozygosity of candidate Hs was thereafter confirmed with microsatellites.

### Seed morphological screen

For seed storage, mesocarps were removed from freshly harvested seed, and seeds air-dried at ambient temperature (24 h). Seeds were thereafter stored at 25°C with 15-18% moisture content. To induce germination, stored seeds were re-hydrated over 3 d to 18-20% moisture content, followed by 38-40°C incubation (40-60 d). Seeds were then re-hydrated for a further 5 d to >22% moisture content, and air-dried at ambient temperature (4 h). Seeds were germinated at ambient temperature (7 d to 3 months after treatment) and examined for atypical germination morphology (Figure 1).

### Molecular pre-screen to exclude heterozygotes

**Figure 7:**
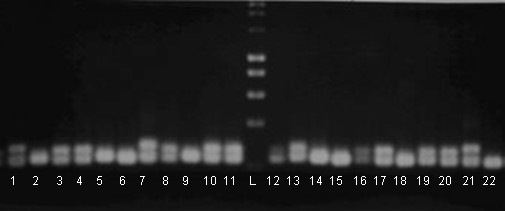
**PCR amplicons generated by microsatellite marker 10 fractionated in 2% w/v agarose**. Lanes 1-11 & 12-20: candidate H/DH palm plants; lane L: HyperladderI (Bioline, UK); lane 21: heterozygote control; lane 22: homozygote control. Candidates in lanes 1, 3, 4, 7, 8, 10, 11, 13, 16, 17, 19, 20 were deemed heterozygous and discarded.

DNA was isolated from leaf tissue using DNeasy 96 Plant Kit (Qiagen, UK). Initial heterozygosity screens used 15 microsatellites (Table 1) yielding alleles readily distinguished by agarose gel electrophoresis (Figure 7). 10 μl PCR mixes comprised 1.0 μl 10× NH_4 _buffer (Bioline), 0.3 μl MgCl_2 _(10 mM), 0.4 μl dNTPs (10 mM), 0.2 μl each primer (10 mM), 1-5 ng DNA and 1U *Taq *polymerase (Bioline). Thermocycling conditions: 2 min at 94°C followed by 35 cycles of 94°C for 30 s, 52-58°C for 30 s and 72°C for 45 s, with a final extension of 72°C for 7 min. Candidates presenting two allelic bands after fractionation by (2-3% w/v metaphor) agarose gel electrophoresis were discarded.

### Extended molecular screen

Candidate DHs and some Hs were subjected to an extensive assay for heterozygosity using 97 fluorescently-labelled microsatellites (Table 3) with 150 seedlings of normal phenotype and 24 heterozygous tenera palms as controls. PCR conditions were as described above and resultant products were fractionated on an ABI3730XL capillary sequencer (Applied Biosystems, USA) by Macrogen Inc (Korea). Allele size was determined (Genemapper v4.0) against a GS400HD standard. Individuals with two alleles at any locus were discarded.

### DH candidate verification

To verify DH candidate 0644-219/05049582C we screened 212 microsatellites (Table 4) for heterozygosity in the maternal parent (BL013/12-06). 10 μl PCR mixes comprising: 5 μl BioMix™(Bioline, UK), 0.05 μl forward primer plus M13 adaptor (10 μM), 0.2 μl labelled M13(-29) (10 μM) (Sigma Genosys, UK), 0.2 μl reverse primer (10 μM) and 5-10 ng DNA were subjected to: 2 min at 94°C, followed by 35 cycles of 30 s at 94°C, 30 s at 52°C, 45 s at 72°C, with a final extension of 72°C for 7 min. Amplicons were surveyed for heterozygosity by high-resolution melt (HRM) analysis according to Croxford *et al. *[26] using the candidate as the reference comparator. Samples with amplicons variable between the maternal parent and candidate DH were fractionated by capillary electrophoresis as above. 48 markers identified as heterozygous in the maternal parent (Table 5) were applied to the DH candidate to assess homozygosity.

**Table 4:** Microsatellite markers used to screen for heterozygosity on the maternal parent (palm BL013/12-06) of DH candidate palm (0644-219/05049582C).

No	Marker	Forward Primer (5'-3')	Reverse Primer (5'-3')
1	VS1	GAGATTACAAAGTCCAAACC	TCAAAATTAAGAAAGTATGC
2	OPSSR 3	ACGCATGCAGCTAGCTTTTC	CGCGTGAAAGATATGAATCAAC
3	OPSSR 7	CACGCACGCAGTTTATTCTT	GGATGTATGCTTTACCTCCGAAT
4	OPSSR 8	CCCCTTTTGCTTCCCTATTT	CTCCTTTTCCCCATCACAGA
5	OPSSR 9	GACACAAGCAAAAACAAAAGCA	ATTCTGAAAGGAGGGGGAAA
6	OPSSR 14	ATATGTGTGGGTGTGCGTGT	TGCCTCTGGTTGTTAGTCTGG
7	OPSSR 19	TCTCTCTCTCTCTCTCTATGTGTGTGT	TGGCAATCAGCACACATTCT
8	OPSSR 29	GCAGCTCTTTCCACACCTCT	TGTGGTCTCCTGAGGAAGATG
9	OPSSR 30	TTTTCCCCATCACAGAATTG	CCCCTTTTGCTTCCCTATTT
10	OPSSR32	GAACAAAACGGGAAGAAGCA	CCTCAAATGGGAGAAACCAG
11	mEgUWA07	CGGATAGAGGCAGCAAGACT	CTCGGGTTGTTTAACCCATT
12	mEgUWA44	TTGAGACGTCGTTCCTTTCC	AGCGGAGACCCAATAATCCT
13	mEgUWA50	CCTGCAACTGCAAATGAGAC	TCCAGACACAAACTACACACACC
14	mEgCIR0037	Published by Billotte *et al. *[27]	
15	mEgCIR0055	Published by Billotte *et al. *[27]	
16	mEgCIR0059	Published by Billotte *et al. *[27]	
17	mEgCIR0067	Published by Billotte *et al. *[28]	
18	mEgCIR0074	Published by Billotte *et al. *[27]	
19	mEgCIR0146	Published by Billotte *et al. *[27]	
20	mEgCIR0163	Published by Billotte *et al. *[27]	
21	mEgCIR0173	Published by Billotte *et al. *[27]	
22	mEgCIR0177	Published by Billotte *et al. *[27]	
23	mEgCIR0192	Published by Billotte *et al. *[27]	
24	mEgCIR0195	Published by Billotte *et al. *[27]	
25	mEgCIR0243	Published by Billotte *et al. *[27]	
26	mEgCIR0246	Published by Billotte *et al. *[27]	
27	mEgCIR0257	Published by Billotte *et al. *[27]	
28	mEgCIR0268	Published by Billotte *et al. *[27]	
29	mEgCIR0328	Published by Billotte *et al. *[27]	
30	mEgCIR0359	Published by Billotte *et al. *[27]	
31	mEgCIR0366	Published by Billotte *et al. *[27]	
32	mEgCIR0369	Published by Billotte *et al. *[27]	
33	mEgCIR0380	Published by Billotte *et al. *[27]	
34	mEgCIR0399	Published by Billotte *et al. *[27]	
35	mEgCIR0408	Published by Billotte *et al. *[27]	
36	mEgCIR0409	Published by Billotte *et al. *[27]	
37	mEgCIR0425	Published by Billotte *et al. *[27]	
38	mEgCIR0433	Published by Billotte *et al. *[27]	
39	mEgCIR0439	Published by Billotte *et al. *[27]	
40	mEgCIR0445	Published by Billotte *et al. *[27]	
41	mEgCIR0446	Published by Billotte *et al. *[27]	
42	mEgCIR0465	Published by Billotte *et al. *[27]	
43	mEgCIR0521	Published by Billotte *et al. *[27]	
44	mEgCIR0551	Published by Billotte *et al. *[27]	
45	mEgCIR0555	Published by Billotte *et al. *[27]	
46	mEgCIR0588	Published by Billotte *et al. *[27]	
47	mEgCIR0772	Published by Billotte *et al. *[27]	
48	mEgCIR0773	Published by Billotte *et al. *[27]	
49	mEgCIR0774	Published by Billotte *et al. *[27]	
50	mEgCIR0775	Published by Billotte *et al. *[27]	
51	mEgCIR0778	Published by Billotte *et al. *[27]	
52	mEgCIR0779	Published by Billotte *et al. *[27]	
53	mEgCIR0781	Published by Billotte *et al. *[27]	
54	mEgCIR0786	Published by Billotte *et al. *[27]	
55	mEgCIR0787	Published by Billotte *et al. *[27]	
56	mEgCIR0788	Published by Billotte *et al. *[27]	
57	mEgCIR0790	Published by Billotte *et al. *[27]	
58	mEgCIR0793	Published by Billotte *et al. *[27]	
59	mEgCIR0800	Published by Billotte *et al. *[27]	
60	mEgCIR0801	Published by Billotte *et al. *[27]	
61	mEgCIR0802	Published by Billotte *et al. *[27]	
62	mEgCIR0803	Published by Billotte *et al. *[27]	
63	mEgCIR0804	Published by Billotte *et al. *[27]	
64	mEgCIR0825	Published by Billotte *et al. *[27]	
65	mEgCIR0827	Published by Billotte *et al. *[27]	
66	mEgCIR0844	Published by Billotte *et al. *[27]	
67	mEgCIR0874	Published by Billotte *et al. *[27]	
68	mEgCIR0878	Published by Billotte *et al. *[27]	
69	mEgCIR0882	Published by Billotte *et al. *[27]	
70	mEgCIR0886	Published by Billotte *et al. *[27]	
71	mEgCIR0894	Published by Billotte *et al. *[27]	
72	mEgCIR0905	Published by Billotte *et al. *[27]	
73	mEgCIR0906	Published by Billotte *et al. *[27]	
74	mEgCIR0910	Published by Billotte *et al. *[27]	
75	mEgCIR0912	Published by Billotte *et al. *[27]	
76	mEgCIR1729	Published by Billotte *et al. *[27]	
77	mEgCIR1740	Published by Billotte *et al. *[27]	
78	mEgCIR1753	Published by Billotte *et al. *[27]	
79	mEgCIR1773	Published by Billotte *et al. *[27]	
80	mEgCIR1917	Published by Billotte *et al. *[27]	
81	mEgCIR1977	Published by Billotte *et al. *[27]	
82	mEgCIR1996	Published by Billotte *et al. *[27]	
83	mEgCIR2110	Published by Billotte *et al. *[27]	
84	mEgCIR2144	Published by Billotte *et al. *[27]	
85	mEgCIR2149	Published by Billotte *et al. *[27]	
86	mEgCIR2188	Published by Billotte *et al. *[27]	
87	mEgCIR2212	Published by Billotte *et al. *[27]	
88	mEgCIR2215	Published by Billotte *et al. *[27]	
89	mEgCIR2380	Published by Billotte *et al. *[27]	
90	mEgCIR2387	Published by Billotte *et al. *[27]	
91	mEgCIR2414	Published by Billotte *et al. *[27]	
92	mEgCIR2417	Published by Billotte *et al. *[27]	
93	mEgCIR2422	Published by Billotte *et al. *[27]	
94	mEgCIR2423	Published by Billotte *et al. *[27]	
95	mEgCIR2427	Published by Billotte *et al. *[27]	
96	mEgCIR2436	Published by Billotte *et al. *[27]	
97	mEgCIR2440	Published by Billotte *et al. *[27]	
98	mEgCIR2492	Published by Billotte *et al. *[27]	
99	mEgCIR2518	Published by Billotte *et al. *[27]	
100	mEgCIR2525	Published by Billotte *et al. *[27]	
101	mEgCIR2569	Published by Billotte *et al. *[27]	
102	mEgCIR2575	Published by Billotte *et al. *[27]	
103	mEgCIR2577	Published by Billotte *et al. *[27]	
104	mEgCIR2590	Published by Billotte *et al. *[27]	
105	mEgCIR2595	Published by Billotte *et al. *[27]	
106	mEgCIR2600	Published by Billotte *et al. *[27]	
107	mEgCIR2621	Published by Billotte *et al. *[27]	
108	mEgCIR2628	Published by Billotte *et al. *[27]	
109	mEgCIR2763	Published by Billotte *et al. *[27]	
110	mEgCIR2813	Published by Billotte *et al. *[27]	
111	mEgCIR2860	Published by Billotte *et al. *[27]	
112	mEgCIR2887	Published by Billotte *et al. *[27]	
113	mEgCIR2893	Published by Billotte *et al. *[27]	
114	mEgCIR3040	Published by Billotte *et al. *[27]	
115	mEgCIR3111	Published by Billotte *et al. *[27]	
116	mEgCIR3160	Published by Billotte *et al. *[27]	
117	mEgCIR3194	Published by Billotte *et al. *[27]	
118	mEgCIR3213	Published by Billotte *et al. *[27]	
119	mEgCIR3232	Published by Billotte *et al. *[27]	
120	mEgCIR3295	Published by Billotte *et al. *[27]	
121	mEgCIR3296	Published by Billotte *et al. *[27]	
122	mEgCIR3297	Published by Billotte *et al. *[27]	
123	mEgCIR3298	Published by Billotte *et al. *[27]	
124	mEgCIR3300	Published by Billotte *et al. *[27]	
125	mEgCIR3301	Published by Billotte *et al. *[27]	
126	mEgCIR3305	Published by Billotte *et al. *[27]	
127	mEgCIR3307	Published by Billotte *et al. *[27]	
128	mEgCIR3310	Published by Billotte *et al. *[27]	
129	mEgCIR3311	Published by Billotte *et al. *[27]	
130	mEgCIR3316	Published by Billotte *et al. *[27]	
131	mEgCIR3321	Published by Billotte *et al. *[27]	
132	mEgCIR3328	Published by Billotte *et al. *[27]	
133	mEgCIR3350	Published by Billotte *et al. *[27]	
134	mEgCIR3384	Published by Billotte *et al. *[27]	
135	mEgCIR3389	Published by Billotte *et al. *[27]	
136	mEgCIR3399	Published by Billotte *et al. *[27]	
137	mEgCIR3400	Published by Billotte *et al. *[27]	
138	mEgCIR3402	Published by Billotte *et al. *[27]	
139	mEgCIR3427	Published by Billotte *et al. *[27]	
140	mEgCIR3428	Published by Billotte *et al. *[27]	
141	mEgCIR3433	Published by Billotte *et al. *[27]	
142	mEgCIR3439	Published by Billotte *et al. *[27]	
143	mEgCIR3477	Published by Billotte *et al. *[27]	
144	mEgCIR3519	Published by Billotte *et al. *[27]	
145	mEgCIR3526	Published by Billotte *et al. *[27]	
146	mEgCIR3533	Published by Billotte *et al. *[27]	
147	mEgCIR3534	Published by Billotte *et al. *[27]	
148	mEgCIR3535	Published by Billotte *et al. *[27]	
149	mEgCIR3538	Published by Billotte *et al. *[27]	
150	mEgCIR3543	Published by Billotte *et al. *[27]	
151	mEgCIR3544	Published by Billotte *et al. *[27]	
152	mEgCIR3546	Published by Billotte *et al. *[27]	
153	mEgCIR3555	Published by Billotte *et al. *[27]	
154	mEgCIR3557	Published by Billotte *et al. *[27]	
155	mEgCIR3563	Published by Billotte *et al. *[27]	
156	mEgCIR3567	Published by Billotte *et al. *[27]	
157	mEgCIR3569	Published by Billotte *et al. *[27]	
158	mEgCIR3574	Published by Billotte *et al. *[27]	
159	mEgCIR3587	Published by Billotte *et al. *[27]	
160	mEgCIR3590	Published by Billotte *et al. *[27]	
161	mEgCIR3592	Published by Billotte *et al. *[27]	
162	mEgCIR3593	Published by Billotte *et al. *[27]	
163	mEgCIR3607	Published by Billotte *et al. *[27]	
164	mEgCIR3622	Published by Billotte *et al. *[27]	
165	mEgCIR3633	Published by Billotte *et al. *[27]	
166	mEgCIR3639	Published by Billotte *et al. *[27]	
167	mEgCIR3643	Published by Billotte *et al. *[27]	
168	mEgCIR3649	Published by Billotte *et al. *[27]	
169	mEgCIR3653	Published by Billotte *et al. *[27]	
170	mEgCIR3655	Published by Billotte *et al. *[27]	
171	mEgCIR3663	Published by Billotte *et al. *[27]	
172	mEgCIR3668	Published by Billotte *et al. *[27]	
173	mEgCIR3672	Published by Billotte *et al. *[27]	
174	mEgCIR3683	Published by Billotte *et al. *[27]	
175	mEgCIR3684	Published by Billotte *et al. *[27]	
176	mEgCIR3691	Published by Billotte *et al. *[27]	
177	mEgCIR3693	Published by Billotte *et al. *[27]	
178	mEgCIR3696	Published by Billotte *et al. *[27]	
179	mEgCIR3698	Published by Billotte *et al. *[27]	
180	mEgCIR3705	Published by Billotte *et al. *[27]	
181	mEgCIR3711	Published by Billotte *et al. *[27]	
182	mEgCIR3716	Published by Billotte *et al. *[27]	
183	mEgCIR3718	Published by Billotte *et al. *[27]	
184	mEgCIR3722	Published by Billotte *et al. *[27]	
185	mEgCIR3727	Published by Billotte *et al. *[27]	
186	mEgCIR3728	Published by Billotte *et al. *[27]	
187	mEgCIR3732	Published by Billotte *et al. *[27]	
188	mEgCIR3737	Published by Billotte *et al. *[27]	
189	mEgCIR3739	Published by Billotte *et al. *[27]	
190	mEgCIR3745	Published by Billotte *et al. *[27]	
191	mEgCIR3747	Published by Billotte *et al. *[27]	
192	mEgCIR3750	Published by Billotte *et al. *[27]	
193	mEgCIR3755	Published by Billotte *et al. *[27]	
194	mEgCIR3766	Published by Billotte *et al. *[27]	
195	mEgCIR3769	Published by Billotte *et al. *[27]	
196	mEgCIR3775	Published by Billotte *et al. *[27]	
197	mEgCIR3782	Published by Billotte *et al. *[27]	
198	mEgCIR3785	Published by Billotte *et al. *[27]	
199	mEgCIR3787	Published by Billotte *et al. *[27]	
200	mEgCIR3788	Published by Billotte *et al. *[27]	
201	mEgCIR3792	Published by Billotte *et al. *[27]	
202	mEgCIR3807	Published by Billotte *et al. *[27]	
203	mEgCIR3808	Published by Billotte *et al. *[27]	
204	mEgCIR3809	Published by Billotte *et al. *[27]	
205	mEgCIR3813	Published by Billotte *et al. *[27]	
206	mEgCIR3819	Published by Billotte *et al. *[27]	
207	mEgCIR3825	Published by Billotte *et al. *[27]	
208	mEgCIR3826	Published by Billotte *et al. *[27]	
209	mEgCIR3828	Published by Billotte *et al. *[27]	
210	mEgCIR3847	Published by Billotte *et al. *[27]	
211	mEgCIR3850	Published by Billotte *et al. *[27]	
212	mEgCIR3869	Published by Billotte *et al. *[27]	

**Table 5:** Markers shown to be heterozygous in the maternal parent (palm BL013/12-06) and homozygous in the DH candidate (0644-219/05049582C).

No	Marker	Linkage Group
1	mEgCIR0268	1
2	mEgCIR0874	1
3	mEgCIR3847	1
4	mEgCIR2149	2
5	mEgCIR2518	3
6	mEgCIR0425	3
7	mEgCIR3544	3
8	mEgCIR3716	4
9	mEgCIR1917	4
10	mEgCIR3535	4
11	mEgCIR3310	4
12	mEgCIR3705	4
13	mEgCIR3477	4
14	mEgCIR0059	4
15	mEgCIR3557	4
16	mEgCIR2813	5
17	mEgCIR3543	6
18	mEgCIR0195	6
19	mEgCIR0894	7
20	mEgCIR0905b	7
21	mEgCIR0774	8
22	mEgCIR2440	8
23	mEgCIR0825	10
24	mEgCIR3826	10
25	mEgCIR0788	10
26	mEgCIR2628	10
27	mEgCIR0146	10
28	mEgCIR0878	11
29	mEgCIR1773	12
30	mEgCIR3311	12
31	mEgCIR0779	14
32	mEgCIR0588	14
33	mEgCIR3737	15
34	mEgCIR3850	15
35	mEgCIR3639	16
36	mEgCIR0905a	16
37	mEgCIR3739	unlinked
38	mEgCIR3160	unmapped
39	mEgCIR3360	unmapped
40	mEgCIR0801	unmapped
41	mEgCIR2577	unmapped
42	OPSSR14	unmapped
43	OPSSR30	unmapped
44	OPSSR32	unmapped
45	mEgUWA44	unmapped
46	mEgUWA50	unmapped
47	mEgUWA07	unmapped
48	VS1	unmapped

Linkage group assigned according to Billotte *et al. *[27].

DH candidate 0644-219/05049582C was found to be homozygous across all 48 loci that were heterozygous in its maternal parent. Of these 48 loci, 36 have been mapped by Billotte *et al. *[27] (Table 5). We first considered the probability of obtaining the observed homozygosity levels via independent assortment using only the unlinked markers from this group. For unlinked loci, the probability of homozygous offspring arising by independent assortment is 0.5 per locus. Given that heterozygous loci were secured from 14 of the 16 linkage groups, with the addition of a further unlinked (unassigned) marker, the probability of these markers all becoming homozygous by chance is therefore: *P *= 0.5^15 ^= 0.000030517578125.

This figure was further reduced by the inclusion of the remaining 21 markers that had been assigned a map position [27]. Here, linkage was accommodated by multiplying by 1-(distance in cM/100). Thus the inclusion of a new marker 10 cM from an existing marker would mean multiplying the cumulative total by 1- (10/100) = 1-0.1 = 0.9 (rather than 0.5 for an unlinked marker). This reduced the probability as follows:

$$\begin{array}{l} P=0.0000\text{3}0\text{517578125}\times \left(\text{extra markers from Linkage Group 1},\text{ LG1}\right)0.\text{92}\times 0.\text{92}\times (\text{extra}\\ \text{markers from LG3})\;0.\text{81}\times 0.\text{93}\times \left(\text{LG4}\right)0.\text{86}\times 0.\text{62}\times 0.\text{55}\times 0.\text{88}\times 0.\text{95}\times 0.\text{87}\times \left(\text{LG6}\right)0.\text{9}\times \\ \left(\text{LG7}\right)0.\text{93}\times \left(\text{LG8}\right)0.\text{52}\times \left(\text{LG1}0\right)0.\text{93}\times 0.\text{94}\times 0.\text{87}\times 0.\text{83}\times \left(\text{LG12}\right)0.\text{5}\times \left(\text{LG14}\right)0.\text{5}\times \\ \left(\text{LG15}\right)0.\text{6}\times \left(\text{LG16}\right)0.\text{51}=\text{8}.\text{72}\times \text{1}0^{-\text{8}}. \end{array}$$

### Flow Cytometry

Newly matured leaflets or radicles from candidate H/DH palms were subjected to flow cytometry according to Anumaganathan & Earle [29] to establish ploidy level. Commercial tenera palms were included as diploid controls. For high-throughput mass screening, tissue samples were bulked at a rate of five individual tissue samples per bulk. Bulked samples (about 0.5 cm^2 ^for radicles and 1 cm^2 ^for leaf material (per each individual) were sliced by chopping with a sharp clean razor-blade (20-30 chops), in a plastic 9 cm diameter Petri dish containing 1.5 ml of cold (5°C) CyStain^® ^UV Ploidy solution (Partec, Germany) modified by addition of 6.48 mM dithiothreitol (DTT) and 1% (v/v) polyvinylpyrrolidone (PVP-40) (Sigma-Aldrich, USA). The addition of DTT and PVP-40 were found to reduce background counts ('noise') in output histograms of particle fluorescence in the analyte.

### Confirmation of Hs by chromosome squashes

Harvested roots were pre-treated in iced water (24 h), then fixed in 3:1 v/v alcohol: glacial acetic acid at 4°C (24 h). They were then rinsed in water, softened in 1N HCl (20 min), rinsed in water (2 min) and stained in saturated aceto-orcein (1 min). The root tip was then squashed, mounted onto a glass slide, and examined using a compound photomicroscope.

### Principal Coordinates Analysis

The genetic affinity of 270 Hs was compared with 95 representative diploids (Table 6) using 28 microsatellites (Table 7) by Principal Coordinates Analysis (PCoA). The PCoA was constructed using GenAlEx v6 [30]. Genetic distance option 'codominant-genotypic' was applied, where pairwise, individual-by-individual (*N × N*) genetic distances are calculated for codominant data. For a single-locus analysis, with *i*-th, *j*-th, *k*-th and *l*-th different alleles, a set of squared distances is defined as *d*^2^(*ii, ii*) = 0, *d*^2^(*ij, ij*) = 0, *d*^2^(*ii, ij*) = 1, *d*^2^(*ij, ik*) = 1, *d*^2^(*ij, kl*) = 2, *d*^2^(*ii, jk*) = 3, and *d*^2^(*ii, jj*) = 4. The algorithm used in GenAlEx is based on Orloci [31] using distance matrix with standardization (by dividing the distance inputs by the square root of *n-1*). Here, Hs were treated as the DHs they were assumed to generate; thus genotypes were homozygous not hemizygous.

**Table 6:** Identification codes, oil palm type and ploidy level of oil palm genotypes used in the Principal Coordinates Analysis

No	Label no in PCO	Sample name in PCO	Palm Id	Ploidy level
1	1	haploid	05020271_0001	x
	
2	2	haploid	05050099_0001	x
	
3	3	haploid	05050099_0002	x
	
4	4	haploid	05020961_0001	x
	
5	5	haploid	05020511_0001	x
	
6	6	haploid	05020946_0001	x
	
7	8	haploid	05030147_0001	x
	
8	9	haploid	05030462_0001	x
	
9	10	haploid	05020420_0002	x
	
10	11	haploid	05020361_0001	x
	
11	12	haploid	05030060_0001	x
	
12	13	haploid	05020558_0001	x
	
13	14	haploid	05020631_0001	x
	
14	15	haploid	05040748_0003	x
	
15	16	haploid	05030308_0001	x
	
16	18	haploid	05080318_0003	x
	
17	19	haploid	06020186_0001	x
	
18	20	haploid	05110212_0001	x
	
19	21	haploid	05120555_0001	x
	
20	22	haploid	06011022_0001	x
	
21	23	haploid	05020059_0001	x
	
22	24	haploid	06020320_0004	x
	
23	25	haploid	06020571_0004	x
	
24	26	haploid	06020381_0001	x
	
25	27	haploid	05060119_0001	x
	
26	28	haploid	05090172_0001	x
	
27	30	haploid	05100321_0001	x
	
28	31	haploid	06010670_0006	x
	
29	32	haploid	06010842_0004	x
	
30	33	haploid	05050228_0001	x
	
31	34	haploid	05110260_0001	x
	
32	35	haploid	05110260_0002	x
	
33	36	haploid	05110162_0001	x
	
34	37	haploid	05101030_0001	x
	
35	38	haploid	05040273_0001	x
	
36	39	haploid	05110003_0001	x
	
37	40	haploid	05120002_0001	x
	
38	41	haploid	05080095_0001	x
	
39	43	haploid	06110122_0002	x
	
40	44	haploid	05110716_0001	x
	
41	45	haploid	05010836_0001	x
	
42	46	haploid	05120155_0001	x
	
43	47	haploid	05110875_0001	x
	
44	48	haploid	05070553_0001	x
	
45	49	haploid	05070466_0001	x
	
46	50	haploid	06010650_0001	x
	
47	51	haploid	05110718_0001	x
	
48	52	haploid	05110496_0001	x
	
49	53	haploid	06010107_0001	x
	
50	54	haploid	05120429_0002	x
	
51	55	haploid	06010953_0001	x
	
52	56	haploid	05030686_0001	x
	
53	57	haploid	05060107_0001	x
	
54	58	haploid	05030791_0001	x
	
55	59	haploid	05080585_0001	x
	
56	60	haploid	05020375_0001	x
	
57	61	haploid	05121048_0001	x
	
58	62	haploid	05055090_0001	x
	
59	63	haploid	05121004_0002	x
	
60	64	haploid	06030064_0001	x
	
61	65	haploid	05121061_0004	x
	
62	66	haploid	05060276_0001	x
	
63	67	haploid	05100988_0001	x
	
64	68	haploid	05060315_0001	x
	
65	69	haploid	06030324_0003	x
	
66	70	haploid	05080506_0001	x
	
67	71	haploid	06010813_0001	x
	
68	72	haploid	05110881_0001	x
	
69	73	haploid	05100717_0001	x
	
70	74	haploid	06020169_0009	x
	
71	75	haploid	05110134_0001	x
	
72	76	haploid	05030196_0001	x
	
73	77	haploid	05050220_0001	x
	
74	78	haploid	06011195_0001	x
	
75	79	haploid	05120725_0001	x
	
76	80	haploid	05100510_0001	x
	
77	81	haploid	05060624_0001	x
	
78	82	haploid	05060712_0001	x
	
79	83	haploid	05030150_0001	x
	
80	84	haploid	06030180_0001	x
	
81	85	haploid	06020915_0001	x
	
82	86	haploid	05101150_0003	x
	
83	87	haploid	05101152_0001	x
	
84	88	haploid	05020415_0001	x
	
85	89	haploid	05040029_0002	x
	
86	90	haploid	05040035_0003	x
	
87	91	haploid	06020573_0001	x
	
88	93	haploid	05121112_0008	x
	
89	94	haploid	05090078_0001	x
	
90	95	haploid	05060495_0001	x
	
91	96	haploid	05070484_0001	x
	
92	97	haploid	06020455_0001	x
	
93	98	haploid	05075185_0001	x
	
94	99	haploid	05090522_0004	x
	
95	100	haploid	06020625_0002	x
	
96	101	haploid	05100812_0002	x
	
97	102	haploid	05100862_0001	x
	
98	103	haploid	05030224_0002	x
	
99	104	haploid	05040439_0001	x
	
100	105	haploid	05040317_0003	x
	
101	106	haploid	05080030_0001	x
	
102	107	haploid	05070703_0003	x
	
103	108	haploid	05080485_0001	x
	
104	109	haploid	05110470_0002	x
	
105	110	haploid	05100423_0001	x
	
106	111	haploid	05110423_0001	x
	
107	112	haploid	05080362_0003	x
	
108	113	haploid	05110625_0001	x
	
109	114	haploid	05120719_0001	x
	
110	115	haploid	05121073_0002	x
	
111	116	haploid	06050726_0002	x
	
112	117	haploid	06060063_0001	x
	
113	119	haploid	06121220_0001	x
	
114	120	haploid	06080516_0001	x
	
115	121	haploid	06090505_0002	x
	
116	122	haploid	06090407_0004	x
	
117	123	haploid	06051133_0002	x
	
118	124	haploid	06060740_0031	x
	
119	125	haploid	06060740_0077	x
	
120	126	haploid	06060740_0090	x
	
121	127	haploid	06120178_0001	x
	
122	128	haploid	06090960_0003	x
	
123	129	haploid	06090657_0001	x
	
124	130	haploid	06120377_0001	x
	
125	131	haploid	06070208_0001	x
	
126	132	haploid	07010308_0001	x
	
127	133	haploid	06121125_0001	x
	
128	134	haploid	06121125_0002 A	x
	
129	135	haploid	06121125_0002 B	x
	
130	136	haploid	06019052_0005	x
	
131	137	haploid	06129197_0001	x
	
132	138	haploid	06079077_0001	x
	
133	139	haploid	07019130_0003	x
	
134	140	haploid	06075474_0001	x
	
135	141	haploid	06075474_0003	x
	
136	142	haploid	06075544_0001	x
	
137	143	haploid	06045801_0001	x
	
138	144	haploid	06065285_0001	x
	
139	145	haploid	06081027_0001	x
	
140	146	haploid	06090264_0001	x
	
141	147	haploid	06090264_0002	x
	
142	148	haploid	06070430_0001	x
	
143	149	haploid	06090861_0001	x
	
144	150	haploid	06051245_0001	x
	
145	151	haploid	06070716_0001	x
	
146	152	haploid	06051468_0001	x
	
147	153	haploid	06075617_0001	x
	
148	154	haploid	06040273_0001	x
	
149	155	haploid	06080584_0001	x
	
150	156	haploid	06070825_0001	x
	
151	158	haploid	06110390_0015	x
	
152	159	haploid	06031385_0001	x
	
153	160	haploid	06045657_0001	x
	
154	161	haploid	06110204_0008	x
	
155	162	haploid	06050161_0001	x
	
156	163	haploid	06071068_0010	x
	
157	164	haploid	06100785_0002	x
	
158	165	haploid	06010987_0028	x
	
159	166	haploid	07010166_0001	x
	
160	167	haploid	06100730_0001	x
	
161	168	haploid	06080681_0001	x
	
162	169	haploid	06080532_0005	x
	
163	170	haploid	06040024_0001	x
	
164	172	haploid	06080217_0010	x
	
165	173	haploid	06120975_0001	x
	
166	174	haploid	06070581_0002	x
	
167	175	haploid	06060477_0001	x
	
168	176	haploid	06120852_0001	x
	
169	177	haploid	06091392_0001	x
	
170	178	haploid	06060344_0001	x
	
171	179	haploid	06090211_0001	x
	
172	180	haploid	06100858_0001	x
	
173	181	haploid	06080272_0007	x
	
174	182	haploid	06050493_0004	x
	
175	183	haploid	06101033_0002	x
	
176	184	haploid	06081043_0001	x
	
177	185	haploid	07011057_0001	x
	
178	186	haploid	06070921_0001	x
	
179	187	haploid	06111210_0002	x
	
180	188	haploid	06121495_0001	x
	
181	189	haploid	06110610_0001	x
	
182	190	haploid	06090772_0001	x
	
183	191	haploid	06090318_0002	x
	
184	192	haploid	06121313_0001	x
	
185	193	haploid	06085027_0001	x
	
186	194	haploid	06090109_0001	x
	
187	195	haploid	06080157_0001	x
	
188	196	haploid	06121316_0001	x
	
189	197	haploid	06110900_0001	x
	
190	198	haploid	06070228_0002	x
	
191	199	haploid	06101174_0001	x
	
192	200	haploid	06060805_0001	x
	
193	201	haploid	06085063_0001	x
	
194	202	haploid	06101037_0001	x
	
195	203	haploid	06110444_0002	x
	
196	204	haploid	06101487_0001	x
	
197	205	haploid	06100937_0001	x
	
198	206	haploid	06090820_0002	x
	
199	207	haploid	06070039_0001	x
	
200	208	haploid	06070772_0001	x
	
201	209	haploid	07011408_0001	x
	
202	210	haploid	07011408_0002	x
	
203	211	haploid	06100319_0001	x
	
204	212	haploid	06070468_0001	x
	
205	213	haploid	06121385_0002	x
	
206	214	haploid	06100537_0001	x
	
207	215	haploid	06120726_0001	x
	
208	216	haploid	06070883_0001	x
	
209	217	haploid	06040041_0001	x
	
210	218	haploid	06100263_0001	x
	
211	219	haploid	06040043_0009	x
	
212	220	haploid	06101232_0001	x
	
213	221	haploid	06060189_0003	x
	
214	222	haploid	06091275_0002	x
	
215	223	haploid	06060097_0001	x
	
216	224	haploid	06100873_0001	x
	
217	225	haploid	06050038_0001	x
	
218	226	haploid	06100025_0001	x
	
219	227	haploid	06100940_0002	x
	
220	228	haploid	06040800_0001	x
	
221	229	haploid	06071007_0002	x
	
222	230	haploid	06020043_0026	x
	
223	231	haploid	06060811_0153	x
	
224	232	haploid	06080751_0001	x
	
225	233	haploid	06050178_0068	x
	
226	234	haploid	06040287_0001	x
	
227	236	haploid	06101496_0001	x
	
228	237	haploid	06040643_0001	x
	
229	238	haploid	06045788_0003	x
	
230	239	haploid	06050326_0001	x
	
231	240	haploid	06080649_0002	x
	
232	241	haploid	06080649_0003	x
	
233	242	haploid	06080601_0001	x
	
234	243	haploid	06101247_0001	x
	
235	244	haploid	06111271_0001	x
	
236	245	haploid	06090337_0001	x
	
237	246	haploid	06050125_0002	x
	
238	247	haploid	06050331_0001	x
	
239	248	haploid	06060728_0002	x
	
240	249	haploid	06080109_0001	x
	
241	250	haploid	06101048_0001	x
	
242	251	haploid	06051077_0001	x
	
243	253	haploid	06041067_0003	x
	
244	254	haploid	06040302_0002	x
	
245	255	haploid	06110121_0001	x
	
246	256	haploid	06090845_0001	x
	
247	257	haploid	06060375_0001	x
	
248	258	haploid	06070494_0001	x
	
249	259	haploid	06040938_0003	x
	
250	260	haploid	06081010_0001	x
	
251	261	haploid	06070415_0003	x
	
252	263	haploid	07010776_0001	x
	
253	264	haploid	06120890_0001	x
	
254	265	haploid	06120316_0001	x
	
255	266	haploid	06121413_0001	x
	
256	267	haploid	06090247_0001	x
	
257	268	haploid	06090247_0002	x
	
258	269	haploid	06090801_0001	x
	
259	270	haploid	06041160_0002	x
	
260	271	haploid	06031248_0001	x
	
261	272	haploid	07010075_0001	x
	
262	273	haploid	07011039_0001	x
	
263	274	haploid	06041232_0001	x
	
264	275	haploid	06101271_0002	x
	
265	276	haploid	06060506_0001	x
	
266	277	haploid	06080566_0001	x
	
267	278	haploid	06060124_0001	x
	
268	279	haploid	07020168_0001	x
	
269	281	haploid	06090909_0002	x
	
270	282	haploid	06080869_0001	x
	
271	1	commercial pisifera	BL605/39-04	2x
	
272	2	commercial pisifera	BL607/91-10	2x
	
273	3	commercial pisifera	BL612/84-05	2x
	
274	4	commercial pisifera	BL1120/75-07	2x
	
275	5	commercial pisifera	BL143/04-10	2x
	
276	6	commercial pisifera	BL147/21-05	2x
	
277	7	commercial pisifera	BL148/05-08	2x
	
278	8	commercial pisifera	BL158/A2-13	2x
	
279	1	commercial tenera	BL10452/207-02	2x
	
280	2	commercial tenera	BL10323/104-06	2x
	
281	3	commercial tenera	BL1177/184-09	2x
	
282	1	commercial dura	BL10887/08-22	2x
	
283	2	commercial dura	BL10885/08-27	2x
	
284	3	commercial dura	BL1221/51-14	2x
	
285	4	commercial dura	BL1222/32-02	2x
	
286	5	commercial dura	BL1224/14-19	2x
	
287	6	commercial dura	BL1231/02-01	2x
	
288	7	commercial dura	BL1235/14-01	2x
	
289	8	commercial dura	BL1125/03-02	2x
	
290	9	commercial dura	BL1124/17-09	2x
	
291	10	commercial dura	BL1136/01-02	2x
	
292	11	commercial dura	BL10868/12-10	2x
	
293	12	commercial dura	BL10868/12-11	2x
	
294	13	commercial dura	BL10868/12-13	2x
	
295	14	commercial dura	BL10879/08-06	2x
	
296	15	commercial dura	BL10879/08-07	2x
	
297	16	commercial dura	BL10879/08-09	2x
	
298	17	commercial dura	BL10883/04-06	2x
	
299	18	commercial dura	BL10883/04-08	2x
	
300	19	commercial dura	BL10883/04-09	2x
	
301	20	commercial dura	BL10883/05-06	2x
	
302	21	commercial dura	BL10891/04-23	2x
	
303	22	commercial dura	BL10891/04-24	2x
	
304	23	commercial dura	BL10891/05-22	2x
	
305	24	commercial dura	BL10891/05-23	2x
	
306	25	commercial dura	BL10873/52-18	2x
	
307	26	commercial dura	BL10873/52-19	2x
	
308	27	commercial dura	BL10873/52-21	2x
	
309	28	commercial dura	BL10873/53-19	2x
	
310	29	commercial dura	BL1229/48-15	2x
	
311	30	commercial dura	BL1230/42-15	2x
	
312	31	commercial dura	A1122/04-01	2x
	
313	32	commercial dura	A1122/12-05	2x
	
314	33	commercial dura	A1122/12-08	2x
	
315	34	commercial dura	A1122/36-02	2x
	
316	35	commercial dura	A1123/01-02	2x
	
317	36	commercial dura	A1123/01-06	2x
	
318	37	commercial dura	A1123/01-07	2x
	
319	38	commercial dura	A1123/01-12	2x
	
320	39	commercial dura	A1130/02-02	2x
	
321	40	commercial dura	A1130/02-06	2x
	
322	41	commercial dura	A1130/02-10	2x
	
323	42	commercial dura	A1130/02-16	2x
	
324	43	commercial dura	A1127/08-16	2x
	
325	44	commercial dura	A1127/08-06	2x
	
326	45	commercial dura	A1127/05-11	2x
	
327	46	commercial dura	A1127/05-03	2x
	
328	47	commercial dura	B1134/35-09	2x
	
329	48	commercial dura	B1133/07-10	2x
	
330	49	commercial dura	B1136/21-11	2x
	
331	50	commercial dura	B1136/21-12	2x
	
332	51	commercial dura	C1128/07-14	2x
	
333	52	commercial dura	C1121/13-08	2x
	
334	53	commercial dura	BL11508/111-1	2x
	
335	54	commercial dura	BL11396/11-21	2x
	
336	1	Ghana wild	K31-1/GHANA/1-1	2x
	
337	2	Ghana wild	K31-1/GHANA/41-498	2x
	
338	3	Ghana wild	K31-1/GHANA/39-875	2x
	
339	4	Ghana wild	K31-1/GHANA/31-430	2x
	
340	5	Ghana wild	K31-1/GHANA/26-629	2x
	
341	6	Ghana wild	K31-1/GHANA/24-1164	2x
	
342	7	Ghana wild	K31-1/GHANA/56-1185	2x
	
343	8	Ghana wild	K31-1/GHANA/29-1087	2x
	
344	9	Ghana wild	K31-1/GHANA/38-1193	2x
	
345	10	Ghana wild	K31-1/GHANA/43-994	2x
	
346	11	Ghana wild	K31-1/GHANA/8-1100	2x
	
347	12	Ghana wild	K31-1/GHANA/11-1192	2x
	
348	13	Ghana wild	K31-1/GHANA/35-1190	2x
	
349	14	Ghana wild	K31-1/GHANA/3-46	2x
	
350	15	Ghana wild	K31-1/GHANA/5-102	2x
	
351	16	Ghana wild	K31-1/GHANA/7-121	2x
	
352	17	Ghana wild	K31-1/GHANA/12-239	2x
	
353	18	Ghana wild	K31-1/GHANA/14-350	2x
	
354	19	Ghana wild	K31-1/GHANA/18-368	2x
	
355	20	Ghana wild	K31-1/GHANA/19-245	2x
	
356	21	Ghana wild	K31-1/GHANA/21-1180	2x
	
357	22	Ghana wild	K31-1/GHANA/32-1141	2x
	
358	23	Ghana wild	K31-1/GHANA/37-1124	2x
	
359	24	Ghana wild	K31-1/GHANA/45-448	2x
	
360	25	Ghana wild	K31-1/GHANA/47-1175	2x
	
361	26	Ghana wild	K31-1/GHANA/50-1037	2x
	
362	27	Ghana wild	K31-1/GHANA/52-547	2x
	
363	28	Ghana wild	K31-1/GHANA/53-1167	2x
	
364	29	Ghana wild	K31-1/GHANA/54-1196	2x
	
365	30	Ghana wild	K31-1/GHANA/57-1153	2x

**Table 7:** Primer pairs used in the Principal Coordinates Analysis to compare the genetic diversity and affinities of Hs compared with a representative sample of commercial and wild diploid palms (listed in Table 6).

No	Primer	Forward (5'-3')	Reverse (5'-3')
1	**1996**	CACTGGGGTCATCTTCATCT	TCGTTCTCTTTCCTTTTGTC

2	**2215**	GAACTTGGCGTGTAACT	TGGTAGGTCTATTTGAGAGT

3	**2427**	GAAGGGGCATTGGATTT	CAGGTGACCAAGTGTAAT

4	**2569**	TAGCCGCACTCCCACGAAGC	CCAGAATCATCAGACTCGGACAG

5	**2595**	TCAAAGAGCCGCACAACAAG	ACTTTGCTGCTTGGTGACTTA

6	**2600**	GGGGATGAGTTTGTTTGTTC	GGCAACATGAAGGTAAG

7	**3282**	GTAACAGCATCCACACTAAC	GCAGGACAGGAGTAATGAGT

8	**3298**	GACTACCGTATTGCGTTCAG	TTTATCAGGAGTTTTTGTTTGAGAG

9	**3311**	AATCCAAGTGGCCTACAG	TCCCTACAATAGCCATCTC

10	**3321**	CAAGGAGGAGCAGGTGAG	TACGGCCTCGGTTCTACAC

11	**3399**	AGCCAATGAAGGATAAAGG	CCACTTAGAGGTAAAACAACAG

12	**3400**	CAATTCCAGCGTFAFTATAG	AGTGGCAGTGGAAAAACAGT

13	**3433**	GGTTCAATGGCATACAT	ACTCCCCTCTTTGACAT

14	**3538**	TCAAGCCACATCCTAACTAC	CTCATAGCCTTTGTTGTGT

15	**3544**	AGCAGGGCAAGAGCAATACT	TTCAGCAGCAGGAAACATC

16	**3546**	GCCTATCCCCTGAACTATCT	TGCACATACCAGCAACAGAG

17	**3574**	AGAGACCCTATTTGCTTGAT	GACAAAGAGCTTGTCACAC

18	**3711**	GTCTCATGTGGCTACCTCTC	GCTAGGTGAAAAATAAAGTT

19	**3819**	CCTCCTTTGGAATTATG	GTGTTTGATGGGACATACA

20	**219**	TTTGCTCGGCGGATACAT	GGAGGGCAGGAACAAAAAGT

21	**257**	GCAGCTAGTCACCTGAAC	GACGAGACTGGAAAGATG

22	**782**	CGTTCATCCCACCACCTTTC	GCTGCGAGGCCACTGATAC

23	**783**	GAATGTGGCTGTAAATGCTGAGTG	AAGCCGCATGGACAACTCTAGTAA

24	**882**	TTGATCTTAGACATAACATACTGTA	AAAGCGCGTAATCTCATAGT

25	**894**	TGCTTCTTGTCCTTGATACA	CCACGTCTACGAAATGATAA

26	**3213**	GCTCTTTGTATTTCCTGGTTC	AGCAGCAAACCCTACTAACT

27	**3691**	GCATCATTGGACTATCATACC	TTGTGAACCAGGGAACTATC

28	**vs1**	GAGATTACAAAGTCCAAACC	TCAAAATTAAGAAAGTATGC

All primers except VS1 were taken from Billotte *et al. *[27].

### Colchicine treatment

Roots of confirmed haploid seedlings were washed and immersed in 2.5, 5.0, 7.5, or 10 mM aqueous colchicine for 5 h. Seedlings were then rinsed with water and planted (2:1:1 v/v compost, sand and soil).

### Cross-fertilization using pollen from H plants

A developing male inflorescence of a confirmed H at the PMC stage was treated with 2.5 mM colchicine *via *injection into the spathe. This treatment was repeated at weekly intervals. The resultant pollen (0.03 g) was applied to a targeted section of the female inflorescence of a diploid dura palm. The inflorescence was then bagged to prevent inadvertent wind pollination.

In addition, some untreated H plants contained up to 30% fully stained pollen using Fluorescein diacetate (FDA) that was presumed to be viable. Pollen from these plants and from palms with apparently inviable pollen (unstained) was applied to targeted sections of a female inflorescence of diploid dura palms in the same way as above.

## Competing interests

JMD, MJW, AEC and CSF have received research funding from BioHybrids International Ltd; SN, SW, ACS, DM and YA are employed fully or in part by Sumatra Bioscience; BPF is contracted to BioHybrids International Ltd; PDSC is Managing Director of BioHybrids International Ltd.

## Authors' contributions

JMD, PDSC, SN and MJW conceived the project. SN, BPF and ACS supervised the phenotypic screen and flow cytometry. MJW supervised the molecular analysis conducted by SW, AEC, CSF and YA, and the cytology conducted by DM. JMD and MJW wrote the manuscript and all authors discussed the results and commented on the manuscript.
